# Associations Between Neighborhood Social Vulnerability and the Distribution of Rhinologists in the United States

**DOI:** 10.1002/ohn.70272

**Published:** 2026-04-30

**Authors:** Asher C. Park, Milan P. Fehrenbach, Raad H. Allawi, Sherefuddin H. Pracha, Oluwatobiloba Ayo‐Ajibola, Kevin Hur

**Affiliations:** ^1^ Feinberg School of Medicine Northwestern University Chicago Illinois USA; ^2^ College of Medicine University of Kentucky Lexington Kentucky USA; ^3^ Keck School of Medicine University of Southern California Los Angeles California USA; ^4^ Department of Otolaryngology–Head and Neck Surgery Keck School of Medicine of the University of Southern California Los Angeles California USA

**Keywords:** rhinology, social determinants of health, Social Vulnerability Index

## Abstract

**Objective:**

Assess the geographic distribution of US rhinologists in relation to neighborhood‐level social determinants of health (SDoH) as measured by the Social Vulnerability Index (SVI).

**Study Design:**

Cross‐sectional.

**Setting:**

United States.

**Methods:**

The American Rhinologic Society directory was queried for US rhinologists and their practice addresses. The distribution of rhinologists was examined at the state and census tract level. SVI scores for each census tract were grouped into four quartiles across Overall SVI and four subthemes: socioeconomic status (SES), household composition and disability status (HH), racial‐ethnic minority status (RE), and housing‐transportation status (HT). The distance from each census tract to the nearest rhinologist was calculated and linear regression assessed associations between distance and SVI.

**Results:**

A total of 808 rhinologists were included in this study. States with the greatest density of rhinologists included Wyoming, Vermont, and the District of Columbia; states with the lowest density included North Dakota, South Dakota, Alaska, and Maine. On multivariable analysis, higher vulnerability in SES (*β* = 1.517 [95% CI: 1.491, 1.544], *P* < .001) and HH (*β* = 1.487 [1.466, 1.509], *P* < .001) was associated with significantly increased distance to the nearest rhinologist. In contrast, higher vulnerability in RE (*β* = 0.347 [0.342, 0.353], *P* < .001) and HT (*β* = 0.926 [0.913, 0.940], *P* < .001) was associated with significantly decreased distance to the nearest rhinologist.

**Conclusion:**

Neighborhood‐level distance to rhinologists in the United States is associated with neighborhood‐level SDoH as measured by SVI. Future studies should assess the impact of rhinologist proximity on health outcomes.

Rhinologists provide care for conditions such as chronic rhinosinusitis, sinonasal tumors, and skull base disorders, which can lead to substantial disease burden if untreated.^1^ Despite the critical nature of this care, prior workforce analyses reveal a significant geographic maldistribution of rhinologists.^2^ Previous studies indicate that most rhinologists are concentrated in urban centers, particularly near academic medical institutions.^3^ This clustering contributes to the creation of “healthcare deserts” in rural and socioeconomically disadvantaged areas.^4^ As a result, patients in underserved regions often face barriers such as long travel distances and delayed access to specialized care, which may exacerbate outcomes in head and neck cancer disease management.^1^, ^2^, ^5^, ^6^, ^7^ This underscores the need for targeted efforts to address the unequal rhinologist distribution and its impact on vulnerable populations.

Existing analyses offer important insights into geographic trends of rhinologists across the United States. Heineman et al conducted a comprehensive workforce analysis of practicing rhinologists nationwide in 2020, identifying significantly unequal geographic distribution in large metropolitan areas such as Los Angeles, Boston, and Manhattan.^3^ Furthermore, this study provided a workforce growth projection by dividing the United States population by the median patient population per hospital referral region, indicating an optimal ratio of one rhinologist per 747,864 patients. The analysis projected a surplus of rhinologists at 1.4 times the optimal workforce size over the next two decades.^3^

Despite this estimated future surplus of rhinologists, Hassanin et al identified geographic clusters of rhinologists primarily along coastal regions, with markedly lower densities in the Central and Midwestern United States.^8^ Their study further highlighted predictors of increased rhinologist density, including regions with higher percentages of non‐citizens, white/Caucasian residents, greater educational attainment, and higher median household incomes.^8^ These findings mirror broader trends in other otolaryngology subspecialty workforce distributions, where factors such as population density, economic resources, and healthcare infrastructure influence provider allocation.^9^, ^10^ While previous studies have shed light on rhinologist distribution in the United States, gaps remain in understanding how these patterns intersect with community‐level social vulnerability as measured by validated social determinants of health (SDoH) indices.

The Social Vulnerability Index (SVI), developed by the Centers for Disease Control and Prevention (CDC), is a widely recognized tool for assessing community‐level vulnerability based on 15 variables grouped into four themes: socioeconomic status (SES), household composition and disability status (HH), racial‐ethnic minority status (RE), and housing‐transportation status (HT).^4^, ^5^, ^11^, ^12^, ^13^ Studies have reported associations between SVI and advanced disease presentation, lower rates of routine surveillance, and worse postoperative outcomes in various pathologies.^4^, ^9^, ^11^, ^14^, ^15^, ^16^, ^17^, ^18^, ^19^ Within otolaryngology, higher SVI scores have been linked to poorer health outcomes and reduced access to specialty care.^11^, ^20^, ^21^, ^22^ By identifying communities with vulnerability across SDoH, the SVI provides a robust framework for analyzing associations between neighborhood‐level SDoH and other factors. A previous study has utilized SVI to examine associations between neighborhood‐level vulnerability and increased distance to the nearest head and neck oncologic surgeon.^23^ Similar application of SVI within the rhinology workforce may identify neighborhoods with heightened vulnerability and increased distance to rhinologists.

This study aims to examine the geographic distribution of rhinologists in the United States and assess its relationship with specific domains of SDoH as measured by SVI. We hypothesize that neighborhoods with higher vulnerability as measured by SVI will be positioned further from rhinologists. Findings from this study have the potential to inform policy interventions, workforce allocation strategies, and educational initiatives in residency and fellowship programs, ultimately supporting efforts to reduce disparities in rhinology access and improve health outcomes for underserved populations.

## Methods

This study was exempt from Northwestern University Institutional Review Board approval as this research did not include human or animal subjects, and all data obtained were publicly available on the internet.

### Identification of the Rhinologist Workforce

The American Rhinology Society (ARS) membership database and the American Academy of Otolaryngology–Head and Neck Surgery (AAO‐HNS) “Find an ENT” directory were primarily utilized to curate the dataset of rhinologists, similar to prior studies.^3^, ^8^, ^23^ The ARS membership database is available online to members of the society and was queried in December 2024; physician names and practice addresses of US‐based rhinologists were extracted from the directory.^24^ The AAO‐HNS “Find an ENT” directory is publicly available online and provides the names, location, and specialty areas of otolaryngologist members of the society.^25^ Rhinologists were selected by querying the “Rhinologist” subspecialty tab of US‐based members within the directory. Members present on both sources were identified to prevent duplication. After querying these resources, states with fewer than five rhinologists were manually queried in Google to identify fellowship‐trained rhinologists that were not included in the ARS and AAO‐HNS databases.

### Social Vulnerability Index

SVI presents ranked scores that serve as percentiles of social vulnerability ranging from 0 to 1, with 0 being the least socially vulnerable and 1 being the most socially vulnerable.^26^ This index is developed from 15 social factors measured by the US Census data and provides ranked scores for Overall SVI and the four subthemes: SES, HH, RE, and HT.^13^, ^27^ The specific social factors associated with Overall SVI and the subthemes are further described in Table 1. Each SVI score describes the social vulnerability of a census tract, which provides neighborhood‐level relative vulnerability in relation to all other census tracts in the United States (Supplemental Figures S1 and S2, available online).

**Table 1:** Overall Social Vulnerability Index Subdivided Into Its Four Subthemes With the Respective Social Factors Comprising Each Subtheme

SVI subthemes	Social factors
Socioeconomic status (SES)	‐ Below 150% poverty
	‐ Unemployed
	‐ Housing cost burden
	‐ No high school diploma
	‐ No health insurance
Household composition and disability status (HH)	‐ Aged 65 and older
	‐ Aged 17 and younger
	‐ Civilian with a disability
	‐ Single‐parent households
	‐ English language proficiency
Racial and ethnic minority status (RE)	‐ Hispanic or Latino (of any race)
	‐ Black or African American^a^
	‐ Asian^a^
	‐ American Indian or Alaska Native^a^
	‐ Native Hawaiian or Pacific Islander^a^
	‐ Two or more races^a^
	‐ Other races^a^
Housing type and transportation (HT)	‐ Multi‐unit structures
	‐ Mobile homes
	‐ Crowding
	‐ No vehicle
	‐ Group quarters

Abbreviation: SVI, Social Vulnerability Index.

^a^ Not Hispanic or Latino.

The practice addresses of all rhinologists included in the study were converted to coordinates and Federal Information Processing Standards (FIPS) codes through the online program, Geocodio.^28^ FIPS codes are unique 11‐digit identifiers that correspond to specific census tracts that were used to link each practice location to its corresponding SVI score from the 2022 Census data available on the CDC website.^26^ Census tracts are geographic subdivisions within a county, used by the US Census Bureau to represent neighborhood‐level population data.

### Distance Mapping and Statistical Methods

SVI scores for each census tract were grouped into four quartiles: “<0.25,” “0.25‐0.4999,” “0.50‐0.7499,” and “0.75‐1.00”, similar to existing practice in SVI research.^29^, ^30^, ^31^, ^32^ The lowest quartile (<0.25) corresponds to areas with the least social vulnerability, while the highest quartile (0.75‐1.00) reflects areas with the highest social vulnerability. For purposes of this study's analysis, the first quartile (lowest vulnerability) served as the reference group.

To analyze proximity to rhinologist locations, the distance from the centroid of each census tract to the nearest rhinologist was calculated and logarithmically transformed to normalize the data distribution. These distances were then assessed across Overall SVI and the subtheme quartiles using univariable linear regression. This was repeated with multivariable linear regression for only the four SVI subthemes to avoid collinearity with Overall SVI. The results of these analyses were exponentiated and expressed as percent change in distance relative to the reference quartile. Descriptive statistics were used to characterize the distribution of rhinologists across Overall SVI and subtheme quartiles and to describe the statewide density of rhinologists across the United States. R Version 4.1.2 (R Foundation) was used for all data processing, distance calculations (in miles), regression analyses, and the creation of choropleth heatmaps.

## Results

### Distribution and Density of Rhinologists by State

**Figure 1:**
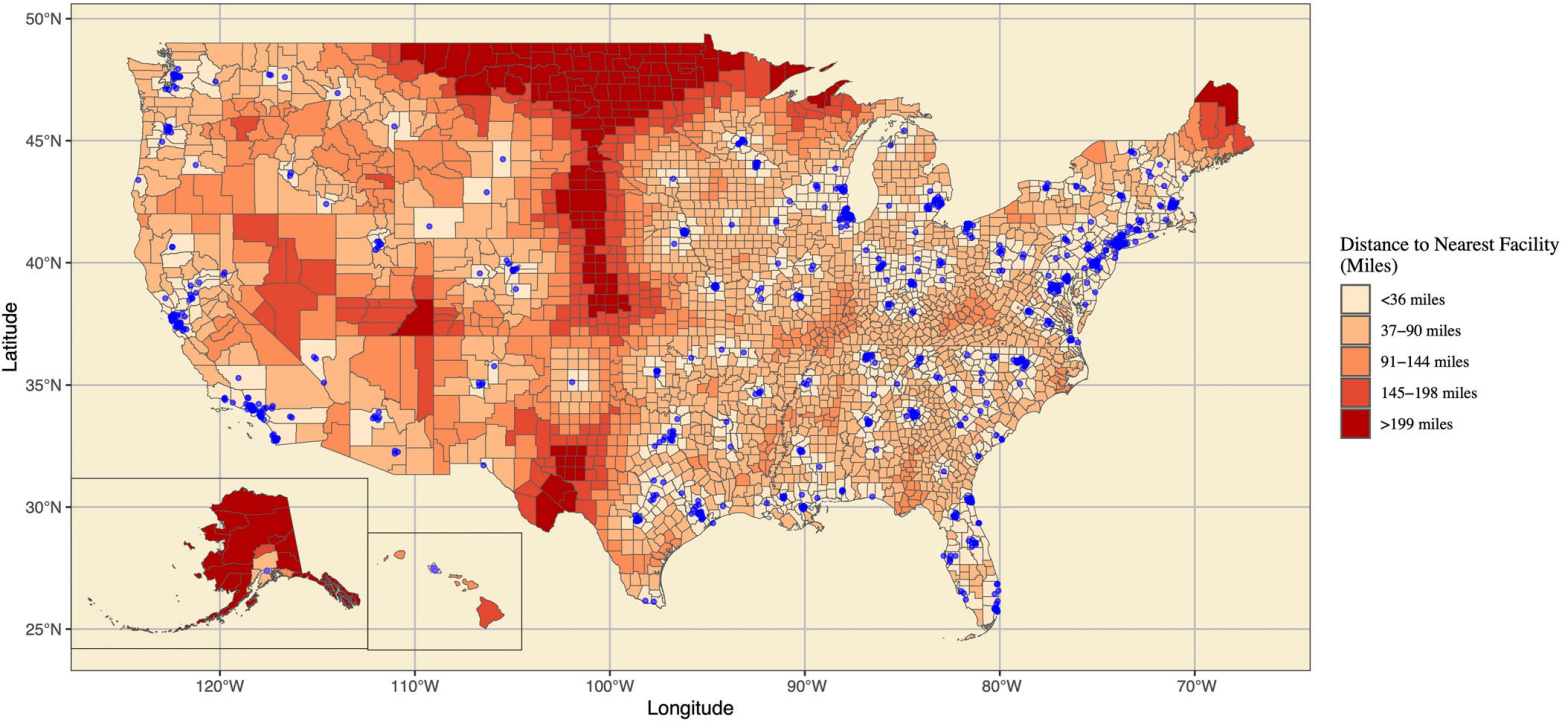
Choropleth heatmap visualizing the distance of the closest rhinologist by county in the United States with blue dots indicating rhinologist practice address.

**Figure 2:**
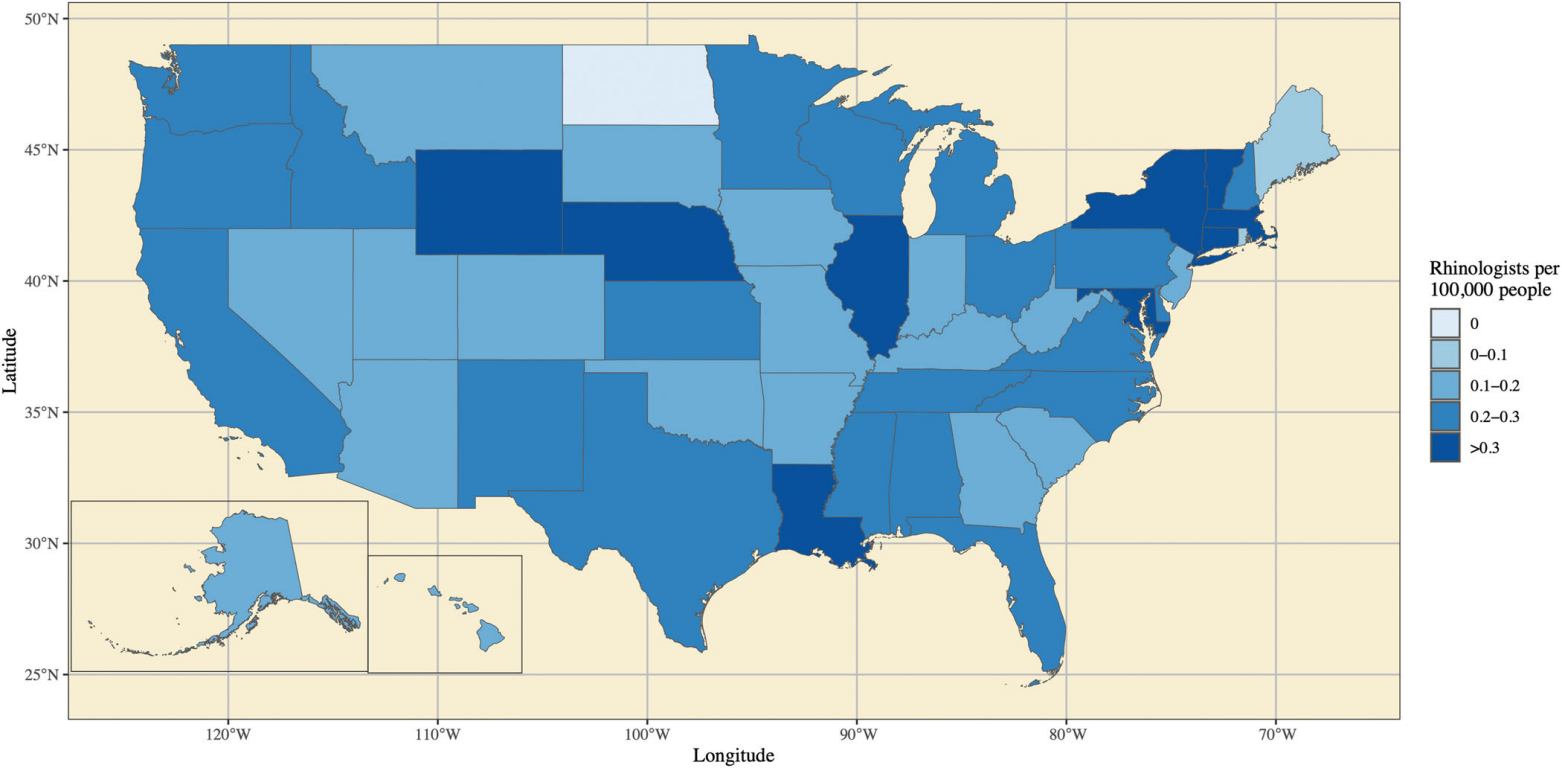
Chloropleth heatmap visualizing rhinologist density per 100,000 population across the United States.

A total of 808 practicing rhinologists were identified across the United States, corresponding to an overall density of 0.241 rhinologists per 100,000 population (Figure 1, Table 2). Regionally, the South (n = 298, 36.9%) contained the greatest density of rhinologists, followed by Western (n = 179, 22.2%), Midwestern (n = 166, 20.5%), and Northeastern (n = 165, 20.4%) regions, respectively. States with the highest absolute number of rhinologists included California (n = 100, 12.4%), Texas (n = 64, 7.92%), and New York (n = 63, 7.80%) whereas the lowest number was identified in North Dakota (n = 0, 0%), Alaska (n = 1, 0.12%), Maine (n = 1, 0.12%), Rhode Island (n = 1, 0.12%), and South Dakota (n = 1, 0.12%). When adjusting for population size, the states with the highest rhinologist density per capita included Vermont (0.463 rhinologists per 100,000 population), Nebraska (0.455 rhinologists per 100,000 population), and the District of Columbia (0.442 rhinologists per 100,000 population) (Figure 2). In contrast, states such as North Dakota (0.00 rhinologists per 100,000 population), Maine (0.072 rhinologists per 100,000 population), and Rhode Island (0.091 rhinologists per 100,000 population) had the lowest rhinologist density.

**Table 2:** Rhinologist Distribution and Density by State

	No. rhinologists	Population	Rhinologist density per 100,000	Relative density to US average
*United States*	808	334,914,895	0.241	1.000
Northeast	165	56,983,517	0.290	1.203
Midwest	166	68,909,283	0.241	1.000
South	298	130,125,290	0.229	0.950
West	179	78,896,805	0.227	0.941
*State*				
Alabama	13	5,108,468	0.254	1.055
Alaska	1	733,406	0.136	0.565
Arizona	10	7,431,344	0.135	0.558
Arkansas	6	3,067,732	0.196	0.811
California	100	38,965,193	0.257	1.064
Colorado	10	5,877,610	0.170	0.705
Connecticut	11	3,617,176	0.304	1.261
Delaware	3	1,031,890	0.291	1.205
District of Columbia	3	678,972	0.442	1.831
Florida	54	22,610,726	0.239	0.990
Georgia	21	11,029,227	0.190	0.789
Hawaii	2	1,435,138	0.139	0.578
Idaho	4	1,964,726	0.204	0.844
Illinois	43	12,549,689	0.343	1.420
Indiana	12	6,862,199	0.175	0.725
Iowa	4	3,207,004	0.125	0.517
Kansas	7	2,940,546	0.238	0.987
Kentucky	7	4,526,154	0.155	0.641
Louisiana	14	4,573,749	0.306	1.269
Maine	1	1,395,722	0.072	0.297
Maryland	26	6,180,253	0.421	1.744
Massachusetts	28	7,001,399	0.400	1.658
Michigan	22	10,037,261	0.219	0.909
Minnesota	13	5,737,915	0.227	0.939
Mississippi	7	2,939,690	0.238	0.987
Missouri	9	6,196,156	0.145	0.602
Montana	2	1,132,812	0.177	0.732
Nebraska	9	1,978,379	0.455	1.886
Nevada	4	3,194,176	0.125	0.519
New Hampshire	4	1,402,054	0.285	1.183
New Jersey	16	9,290,841	0.172	0.714
New Mexico	5	2,114,371	0.236	0.980
New York	63	19,571,216	0.322	1.334
North Carolina	27	10,835,491	0.249	1.033
North Dakota	‐	783,926	‐	‐
Ohio	33	11,785,935	0.280	1.161
Oklahoma	6	4,053,824	0.148	0.613
Oregon	11	4,233,358	0.260	1.077
Pennsylvania	37	12,961,683	0.285	1.183
Rhode Island	1	1,095,962	0.091	0.378
South Carolina	8	5,373,555	0.149	0.617
South Dakota	1	919,318	0.109	0.451
Tennessee	21	7,126,489	0.295	1.221
Texas	64	30,503,301	0.210	0.870
Utah	6	3,417,734	0.176	0.728
Vermont	3	647,464	0.463	1.921
Virginia	19	8,715,698	0.218	0.904
Washington	20	7,812,880	0.256	1.061
West Virginia	2	1,770,071	0.113	0.468
Wisconsin	12	5,910,955	0.203	0.841
Wyoming	3	584,057	0.514	2.129

### Rhinologists by SVI Quartiles

Within Overall SVI, rhinologists were relatively evenly distributed across all four quartiles of Overall SVI scores (Table 3). Similarly, rhinologist distribution was relatively uniform across quartiles of SES subtheme scores. In contrast, the majority of rhinologists resided in census tracts within the first quartile of HH subtheme scores. For the RE and HT subthemes, most rhinologists were located in census tracts within the third and fourth quartiles of scores, respectively.

**Table 3:** Rhinologist Distribution by Social Vulnerability Index (SVI) Quartile

SVI theme	Frequency, %
Overall SVI	
1st quartile	155 (19.5)
2nd quartile	205 (25.9)
3rd quartile	222 (28.0)
4th quartile	211 (26.6)
Socioeconomic status SVI	
1st quartile	230 (29.0)
2nd quartile	153 (19.3)
3rd quartile	198 (25.0)
4th quartile	212 (26.7)
Household composition and disability status SVI	
1st quartile	344 (43.4)
2nd quartile	161 (20.3)
3rd quartile	157 (19.8)
4th quartile	131 (16.5)
Racial‐ethnic minority SVI	
1st quartile	81 (10.0)
2nd quartile	207 (25.7)
3rd quartile	323 (40.1)
4th quartile	195 (24.2)
Housing transportation SVI	
1st quartile	73 (9.2)
2nd quartile	99 (12.5)
3rd quartile	168 (21.2)
4th quartile	453 (57.1)

### Associations Between SVI and Distance to Nearest Rhinologist

Associations between Overall SVI, SES, HH, RE, and HT scores and distance (miles) to the nearest rhinologist were evaluated through univariable and multivariable linear regression analysis (Table 4). On univariable analysis, there was a 2% decrease in distance to the nearest rhinologist as social vulnerability increased from the least to most vulnerable quartiles (*β* = 0.984, [95% CI: 0.970‐0.998], *P* = .022). When adjusting for SVI subthemes on multivariable analyses, there was a 52% increase in distance to the nearest rhinologist from the least to most vulnerable SES subtheme quartiles (*β* = 1.52, [95% CI: 1.491‐1.544], *P* < .001). Similarly, distances to the nearest rhinologist increased by 49% as HH vulnerability increased from the least to most vulnerable quartiles (*β* = 1.487, [95% CI: 1.466‐1.509], *P* < .001). Conversely, there was a 66% decrease in distance to the nearest rhinologist across the RE subtheme from the least to most vulnerable quartiles (*β* = 0.347, [95% CI: 0.342‐0.353], *P* < .001). Similarly, as vulnerability in the HT subtheme increased from the least to most vulnerable quartiles, distance to the nearest rhinologist decreased by 8% (*β* = 0.940, [95% CI: 0.913‐0.940], *P* ≤ .001).

**Table 4:** Univariable and Multivariable Linear Regression Analysis of Rhinologist Proximity Across Quartiles of Social Vulnerability Index (SVI), With the First Quartile Serving as the Reference Category

	Univariable		
Characteristic	Coefficient	95% CI	*P*‐value
Overall SVI	0.984	0.970, 0.998	.022
Socioeconomic status SVI theme	1.017	1.003, 1.031	.019
Household composition and disability status SVI theme	1.242	1.225, 1.260	<.001
Racial‐ethnic minority SVI theme	0.490	0.483, 0.496	<.001
Housing transportation SVI theme	0.967	0.954, 0.981	<.001

	Multivariable		
Characteristic	Coefficient	95% CI	*P*‐value
Socioeconomic status SVI theme	1.517	1.491, 1.544	<.001
Household composition and disability status SVI theme	1.487	1.466, 1.509	<.001
Racial‐ethnic minority SVI theme	0.347	0.342, 0.353	<.001
Housing transportation SVI theme	0.926	0.913, 0.940	<.001

## Discussion

This study provides a unique overview of the geographic distribution of rhinologists across the United States, identifying neighborhood‐level disparities in distance to rhinologists based on SDoH as measured by the SVI. Our analysis demonstrates that census tracts with greater vulnerability across SES and HH subthemes were associated with significantly increased distances to the nearest rhinologist. Conversely, census tracts with greater vulnerability across RE and HT were associated with decreased distances to the nearest rhinologist. These findings contribute to the growing body of literature on specialty healthcare workforce distributions and emphasize the need for investigation of the clinical implications of increased distance to rhinology subspecialists.

Currently, the literature assessing associations between rhinologist accessibility and health outcomes is limited. A large multi‐institutional study evaluated the association between distance traveled by patients and severity of chronic rhinosinusitis symptoms but found no significant correlations.^33^ However, this represents only one pathology managed by rhinologists, and conclusions regarding the association between distance to rhinologists and outcomes across other pathologies cannot be extrapolated. Despite the sparsity of rhinology literature on this topic, other otolaryngology subspecialties have demonstrated clinically significant associations between provider distance and patient outcomes. Previous studies evaluating head and neck cancer patients revealed that increased distances to the nearest hospital were associated with increased stage at presentation, higher rates of surgery, and decreased follow‐up length and frequency.^7^, ^34^, ^35^ It is possible that similar disparities exist across sinonasal and/or skull base cancers, and future studies should investigate this paucity in the literature. Similar associations have also been reported across other pathologies outside of otolaryngology, underscoring that even modest changes in distance may still significantly affect a patient's disease burden. For instance, Borren et al found that patients with inflammatory bowel disease had a 68% increased odds of requiring surgical intervention when living 8.8 miles away from the hospital, in comparison to those living 2.5 miles away.^36^ Ultimately, our study has revealed significant associations between neighborhood‐level SDoH and distance to rhinologists; further studies should be conducted to assert the clinical impact of these geographic disparities across the United States.

The distribution of rhinologists reveals significant geographic variability, with marked differences in density across states. The Northeastern United States exhibited the highest rhinologist density, with notable states such as Connecticut, Massachusetts, New Hampshire, New York, Pennsylvania, and Vermont exceeding the national average of 0.241 rhinologists per 100,000. This aligns with findings among existing studies on the distribution of other otolaryngology subspecialists and other medical specialists, wherein coastal and densely populated regions often have higher physician densities due to the regionalization of academic centers and large patient populations.^37^, ^38^, ^39^ These distribution patterns are consistent with the findings of Talwar et al, who reported that the District of Columbia, New York, and Massachusetts were among the ten states most densely populated with head and neck surgeons.^9^ Similarly, Sannes et al described the geographic distribution of facial plastic surgeons, noting a predominance of surgeons in the 10 most populated metropolitan areas including northeastern cities such as New York and the District of Columbia.^40^ There are further similarities between our own study findings and those of existing literature. Talwar et al found the Great Plains region reportedly had fewer head and neck surgeons, aligning with our analysis as both North and South Dakota had either one or no practicing rhinologists.^9^ This underscores the persistence of geographic gaps in otolaryngology subspecialty care across the United States, a disparity potentially influenced by fellowship training and a predominance of teaching hospitals and healthcare systems in metropolitan areas.^41^ Recent ARS survey data suggest that 74% of rhinology fellows obtain academic positions following training, which may further concentrate subspecialists in tertiary academic centers.^42^

Utilizing a multivariate analysis across SVI subthemes to assess the geospatial distribution of rhinologists helps delineate the specific social themes associated with the greatest changes in distance to the nearest rhinologist. In our study, census tracts in the most vulnerable quartile of SES were associated with the greatest distance to the nearest rhinologist, followed by those in the most vulnerable quartile of HH. These findings support the notion that patients located further from healthcare centers are likely to have a lower SES and greater housing vulnerability.^43^ Similar results have been reported across other subspecialties, where census tracts with higher SES and HH scores were associated with significantly increased distances to the nearest head and neck oncology surgeon.^23^ Furthermore, greater SES and HH vulnerability have been associated with an increased risk of postoperative complications, such as surgical wound infection and re‐bleeding following pediatric tonsillectomies, suggesting that patients in these communities not only live further from healthcare facilities but may also face worse health outcomes.^44^, ^45^ This theme is reinforced in studies associating lower patient SES with delays in presentation of sinonasal and paranasal sinus malignancies.^46^, ^47^ Similar trends were observed among sinonasal cancer patients with increased HH vulnerability, which has been associated with decreased surveillance, follow‐up, and survival.^48^ Increased distances to rhinologists may be a contributing factor that compounds adverse health outcomes among patients living in regions with greater SES and HH vulnerability, but further studies are required to discern if such a relationship exists.

Contrasting SES and HH subthemes, the RE subtheme demonstrated an inverse relationship; as neighborhood‐level vulnerability across these themes increased, there was a significant decrease in distance to the nearest rhinologist. The variable distribution of ethnic minority populations across geographic settings may be a factor contributing to these findings.^49^ United States census data has indicated that roughly 64% of individuals residing in metropolitan cities identify as Hispanic, black, Asian, and other non‐white ethnicities.^49^ In contrast, white individuals account for a larger proportion of rural populations (78.2%) compared to urban populations (57.3%).^50^ This demographic distribution aligns with otolaryngologist practice patterns, as a previous study evaluating medicare utilization and reimbursement variation among otolaryngologists found that 92% practiced in urban settings.^51^ When stratified by subspecialty, larger proportions of otology‐neurotology, head and neck, and facial plastic surgeons have been shown to reside mostly in densely populated metropolitan areas as well.^9^, ^10^, ^40^, ^52^ This geographic clustering suggests that racial and ethnic minority communities are closer in distance to rhinologists, which is reflected by the RE subtheme in this analysis. Nevertheless, increased rhinologist proximity may not necessarily result in improved accessibility or utilization of such services. Disparities in healthcare utilization have been well‐documented with insurance status limiting access to complex surgical care among minority groups.^53^, ^54^ In addition to limitation in acquiring complex treatment, delays in presentation have also been reported. For instance, a recent SEER database study found greater prevalences of distant‐stage cancer diagnosis among non‐insured racial and ethnic minority groups.^55^ Cultural factors may also limit the use of medical services as minority groups have shown a preference for receiving care from racially concordant physicians.^56^ Overall, racial and ethnic minority groups appear to reside closer to rhinologists; however, systemic barriers may affect accessibility despite this proximity. Similarly, census tracts with increasing vulnerability across the HT subtheme were associated with proximity to the nearest rhinologist. Similar to the findings within the RE subtheme, this may be because areas with greater vulnerability within the HT subtheme tend to be in metropolitan areas. Factors that comprise the HT subtheme include multi‐unit housing structures, group housing, crowding, lack of personal vehicle, and mobile homes. With the exception of the latter, these social factors within SVI's housing‐transportation subtheme intuitively tend to be more prevalent within metropolitan areas where rhinologists have been shown to practice as previously mentioned and likely contribute to our findings.

This study is subject to several limitations. The cohort of rhinologists in this study was limited to members of the ARS database and AAO‐HNS directory, which likely are not inclusive of all actively practicing rhinologists in the United States. In an effort to remediate this discrepancy, states with fewer than five rhinologists were manually queried to expand the cohort. Additionally, the distances to the nearest rhinologist were measured from the center of each census tract. This measurement may not be precisely representative of the distance traveled to the nearest rhinologist and additionally may not represent the rhinologist that patients are able to see.

## Conclusion

This study reveals significant geographic disparities in the distribution of rhinologists across neighborhood‐level SDoH, with increased distances to rhinologists seen in neighborhoods with greater SES and household composition and disability status vulnerability. In contrast, communities with higher vulnerability amongst RE and HT were located closer to rhinologists. Future research should explore how these geographic disparities translate into differences in clinical outcomes, healthcare utilization, and patient satisfaction in order to better direct strategic interventions to improve access to rhinologic care and patient outcomes.

## Author Contributions

**Asher C. Park**, conceptualization, methodology, data curation, formal analysis, investigation, writing—original draft, writing—review and editing; **Milan P. Fehrenbach**, conceptualization, methodology, data curation, formal analysis, investigation, writing—original draft, writing‐review and editing; **Raad H. Allawi**, conceptualization, methodology, data curation, formal analysis, investigation, writing—original draft, writing—review and editing; **Sherefuddin H. Pracha**, conceptualization, methodology, data curation, formal analysis, investigation, writing—original draft, writing—review and editing; **Oluwatobiloba Ayo‐Ajibola**, conceptualization, methodology, data curation, formal analysis, investigation, writing—original draft, writing—review and editing; **Kevin Hur**, conceptualization, methodology, data curation, formal analysis, investigation, writing—original draft, writing—review and editing, supervision.

## Disclosures

### Competing interests

The authors declare no conflicts of interest.

### Funding source

The authors received no financial support for the research, authorship, and/or publication of this article.

## Supporting information

Supporting Information.

Supporting Information.
