# Supplementary figures and images for: Associations Between Neighborhood Social Vulnerability and the Distribution of Rhinologists in the United States
Source: Otolaryngol Head Neck Surg. 2026 Apr 30;175(1):61–70. doi: 10.1002/ohn.70272 (PMC13327424; doi:10.1002/ohn.70272)

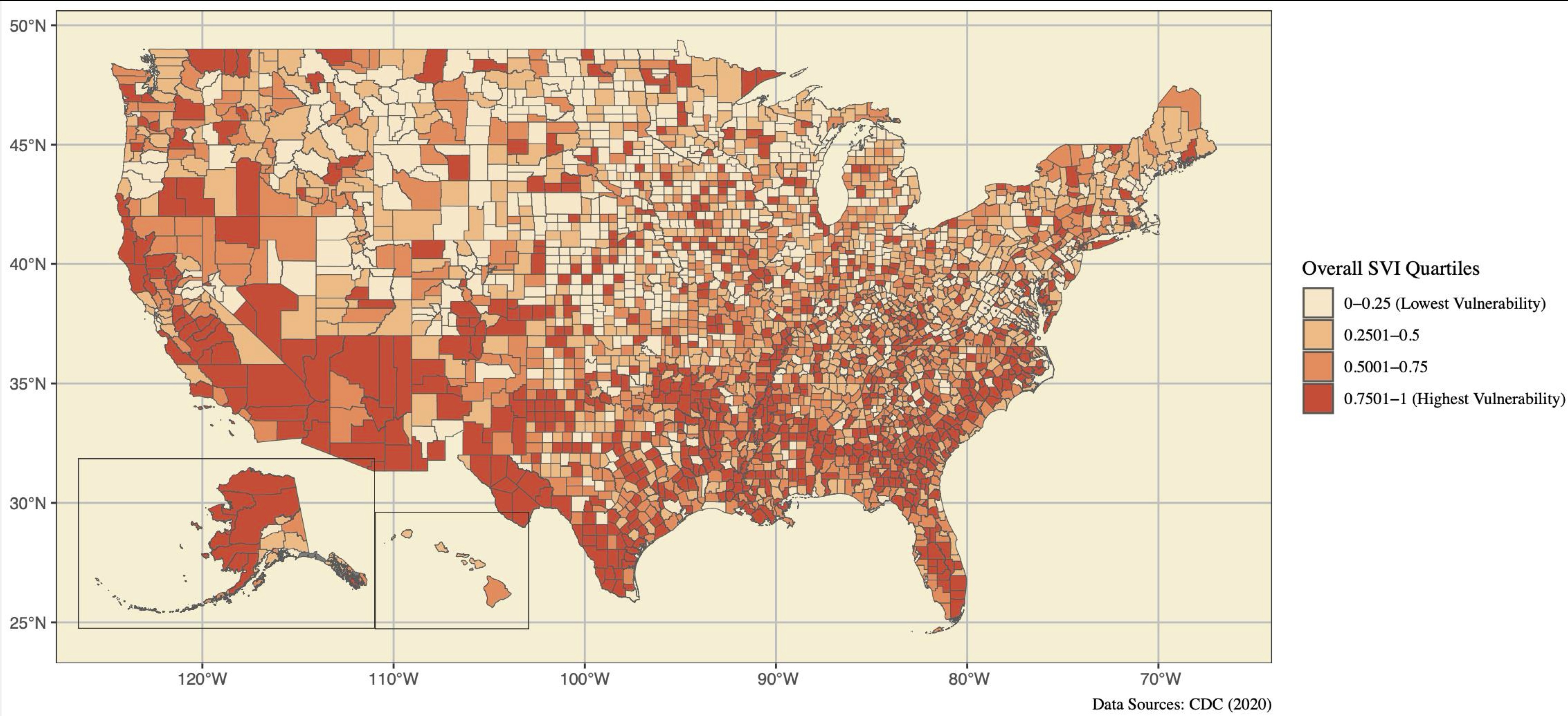

Supplement: Supplementary file 1 — Supporting Information. [file OHN-175-61-s001.jpg]

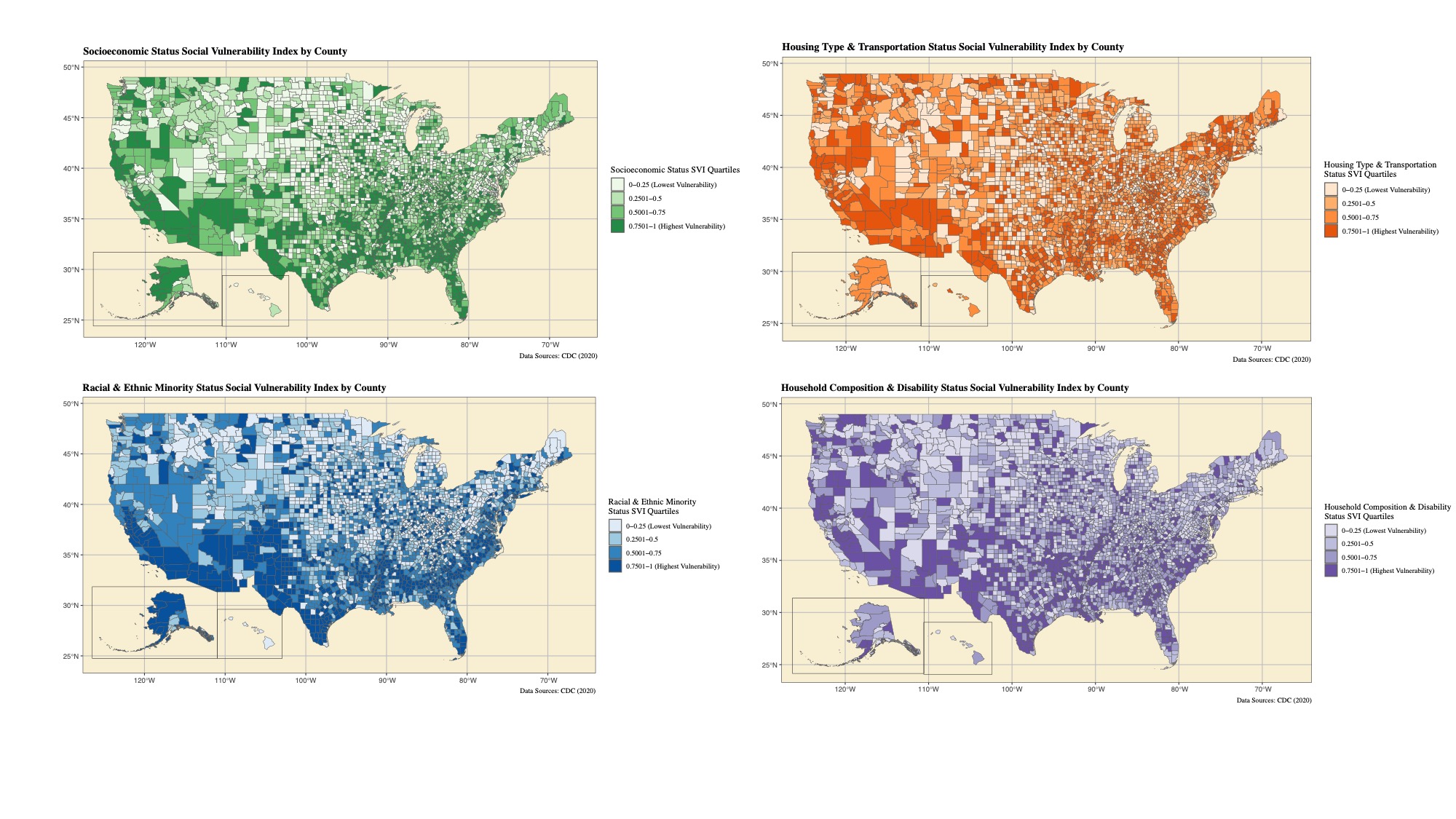

Supplement: Supplementary file 2 — Supporting Information. [file OHN-175-61-s002.jpg]
